# Objectively measured physical activity in two-year-old children – levels, patterns and correlates

**DOI:** 10.1186/s12966-015-0161-0

**Published:** 2015-01-24

**Authors:** Elin Johansson, Maria Hagströmer, Viktoria Svensson, Anna Ek, Michaela Forssén, Håkan Nero, Claude Marcus

**Affiliations:** Karolinska Institutet, Department of Clinical Science, Technology and Intervention, Division of Pediatrics, Stockholm, Sweden; Karolinska Institutet, Department of Neurobiology, Care Sciences and Society, Division of Physiotherapy, Stockholm, Sweden; Karolinska University Hospital at Huddinge, Barnendokrinlab B62, SE-141 86 Huddinge, Sweden

**Keywords:** Accelerometer, BMI, Energy expenditure, Motor skills, Toddler

## Abstract

**Background:**

The aim was to describe levels, patterns and correlates of physical activity and sedentary behavior in a sample of Swedish children, two years of age, with normal weight, overweight and obese parents.

**Methods:**

Data from 123 children, 37 with normal-weight parents and 86 with overweight/obese parents, enrolled in the Early Stockholm Obesity Prevention Project study was used. Children wore an Actigraph GT3X+ accelerometer for seven days. Average activity (counts per minute), number of steps and time spent in low and high-intensity physical activity and in sedentary was assessed. Differences between weekdays and weekend days were examined as were correlations with sex, body mass index (BMI), motor skills and family-related factors.

**Results:**

Children were active at high intensity 11% of the day. On average 55% of the day was spent being sedentary. Number of steps and time in low-intensity physical activity differed between weekdays and weekend days: on weekdays, 363 more steps (p = 0.01) and six more minutes in low physical activity (p = 0.04). No differences were found for any physical activity or sedentary behavior variable by sex, BMI, motor skills or any family-related variable (p = 0.07 – 0.95).

**Conclusions:**

Two-year-old children have an intermittent activity pattern, that is almost similar on weekdays and they spend about half of the daytime active. The absence of any association with sex, BMI, motor skills or parental factors indicates that the individual variation in this age group is primarily due to endogenous factors.

**Trial registration:**

Clinicaltrials.gov: NCT01198847.

## Background

Physical activity (PA) affects health in both children and adults [1,2] and it is possible that PA improves health already at four years of age [3]. Little is known about levels, patterns and correlates of PA and sedentary behavior (SB) in children under the age of three and preschool children are generally assumed to be habitually active [4] but this has not been confirmed by research. Several factors, such as sex [5,6], adiposity [6-8] and motor skills [9,10] have been associated with PA and SB in school-aged children from at least six years of age but it is not clear at what age these associations start to occur. In young children, parents play an important role for health-related behaviors [11]. Thus, family-related factors such as parental education [12,13], parental body mass index (BMI) [12] and siblings [12] might affect young children’s PA and SB.

PA is a complex and multidimensional behavior which makes it difficult to assess. Objective methods are preferable [14] and accelerometers are often used to assess PA and SB in school-aged children and adults. The use among preschoolers is increasing; however studies of objectively measured PA and SB over several days in children under the age of three are still sparse. Accelerometers can be worn on different body locations, such as the waist, wrist or ankle [15,16]. These devices are also designed to measure sleep [17], and a wrist placement is more feasible if the monitor is worn during night time. A wrist placement might also increase compliance.

The Early Stockholm Obesity Prevention Project (Early STOPP) is an ongoing cluster randomized controlled trial including children with high and low risk of becoming overweight or obese, based on parental BMI [18]. The Early STOPP cohort has a longitudinal design and enables studies of obesity risk factors, such as motor skills and objectively measured PA and SB.

The aim of the present study was to describe the levels and patterns of PA and SB in a sample of Swedish children, two years of age. A further aim was to study possible correlates of PA and SB such as sex, BMI, motor skills and family-related factors.

## Methods

### Participants

Two-year follow-up data from the Early STOPP study was used. Families were recruited based on parental BMI (at least one parent with BMI ≥ 30 or two parents with BMI ≥ 25) from pediatric health care centers in Stockholm before the child’s first birthday. Additionally, a reference group of families with normal-weight parents were enrolled in the study, representing a low risk group. Children with chronic health problems likely to influence growth, physical activity, or eating habits had been excluded prior to the baseline visit. High risk families were allocated to either intervention or control group, through cluster randomization of pediatric health care centers. Families are followed yearly until the child’s sixth birthday. Recruitment started in 2010 and was completed in early 2013. The Early STOPP study was approved by The Stockholm Regional Ethical Review Board (2009/217-31/2) and families signed forms giving their informed consent prior to their inclusion. Detailed information about the study can be found elsewhere [18,19]. In total 178 children (51 with normal-weight parents and 127 with either two parents being overweight or at least one parent being obese) attended the two-year follow-up visit in the Early STOPP study and were avaliable for inclusion in this sub-study.

### Physical activity and sedentary behavior

PA and SB were assessed using the Actigraph GT3X+ accelerometer (Actigraph, Pensacola, FL), a 5 × 5 × 2 cm monitor, which is lightweight and water-resistant. The accelerometer was mailed to the families, along with detailed information on how to use the accelerometer. Children wore the accelerometer, attached by a strap, on their left wrist for seven consecutive days and nights [20]. After the measurement period the accelerometers were collected by research staff at the two-year visit.

A sampling rate of 30 Hz was used and accelerometer data was tallied at five-second intervals, as suggested in order to capture the short bursts of activity that is characteristic of young children [21]. The accelerometer data, vertical axis and vector magnitude (VM), were downloaded and analyzed in the ActiLife program, version 6.8 (Actigraph, Pensacola, FL). A pragmatic approach was used to remove sleep time. The hours between 8 p.m. and 7 a.m. were excluded from the analysis, since two-year old children usually go to bed between 8 and 9 p.m. and rise between 7 and 8 a.m. [22,23]. Sleep during the day was considered sedentary time. Children with a minimum of four days of data, including at least one weekend day, were included [24].

Outcome variables were average PA expressed as counts per minute (CPM) for the vertical axis and the VM. Accelerometer steps have not been validated in preschool children but were used as a proxy for overall activity. Time spent in different intensities was also assessed. Minutes per day spent in SB and in low and high-intensity PA was calculated based on intensity thresholds developed by our group [25]. Accelerometer counts ≤89 and ≥440 per five seconds for the vertical axis were used for sedentary and high-intensity PA, respectively. In addition, to study behavioral patterns, bouts of PA and SB were calculated. For time spent in low and high-intensity PA bouts of five minutes were used. For each five-minute bout of low-intensity, activity below 89 counts and exceeding 440 counts per 5 minutes was accepted for one minute. For each five-minute bout in high-intensity PA, one minute below 440 was accepted for one minute. For SB, bouts of 30 minutes, allowing one minute of counts exceeding 89 counts per 5 second, were computed. Data for weekdays and weekend days were examined separately in order to study the pattern over the week.

### Height and weight

Height was measured using a stadiometer and weight was measured, using a portable scale (Tanita HD-316, Tanita Corp.; Tokyo, Japan) in both children and parents. BMI (kg/m^2^) was calculated and weight status determined (normal, overweight or obese). For children, BMI was classified according to Cole et al [26]. In children the categories overweight and obese were merged since only one child was considered obese. To calculate the BMI standard deviation score (BMI SDS) Swedish sex and age specific reference values were used [27].

### Motor skills

Motor skills were assessed using the “neurological examination technique for toddler-age” according to Hempel [28]. This test, which is intended to detect minor differences in motor skill development, is designed for children of 1.5-four years of age and has shown satisfactory inter-rater reliability (kappa value between 0.62–1.0 for the items, mean value 0.93). The test takes about 30 minutes to perform and includes assessment of fine-, gross-, and fundamental movement skills. The test consists of seven parts; prehension, sitting, crawling, standing, walking, and assessment of cranial nerve function and sensomotor function (e.g. assessment of muscle tone and reflexes). A manual is provided that describes in detail how items are to be assessed and interpreted. The test was carried out by one of two alternating physiotherapists, trained to perform and assess the test. The test was video recorded and the video was then watched so that items could be scored.

Items are summarized in a Neurological Optimality Score (NOS) ranging from 0-58. In addition to the total score, the children were classified into NOS <53 and ≥53, in order to compare those given lower scores with those given higher scores. Since no reference values for what might be “low” or “high” NOS were provided, the dichotomy variable was computed based upon a previous study showing that the median NOS for children exposed to pre- and postnatal polychlorinated biphenyls and dioxins was 53 [29]. More information about the test can be found elsewhere [28].

### Family-related factors

Data on older siblings, child care and parental education were collected via questionnaires filled in by the parents. The children attended preschool, full-time (≥30 h/day) or part-time (<30 h/day), or were in other types of child care (e.g. were taken care of by a parent or babysitter). Regarding educational level there were three response options: nine years of school, 12 years of school, more than 12 years of school. The educational levels of the parents were combined and dichotomized into high and low parental educational levels. If one or both parents had more than 12 years of school the family was considered as having a high level of education.

### Statistical analysis

Since no differences in outcome measures were seen between intervention and control families at the 2-year visit the groups were merged and referred to as the high risk group. Descriptive data on age, BMI, BMI SDS and NOS score were presented as means and standard deviations (SD). For sex, weight status (normal/overweight), NOS < and ≥53, classification into family group (high-risk/low-risk), first-born status, child care situation (full-time or part-time preschool/other) and parental education (high/low), n (%) was used. For comparisons of background characteristics between children who wore and children who did not wear the accelerometer, an independent t-test was used.

All PA and SB variables were presented as mean (SD) and were also calculated as means (SD) as a weekly average and for weekdays and weekend days separately. To assess differences in PA and SB between weekdays and weekend days, the paired samples t-test was used and Cohen’s d was calculated. Cohen’s d 0.2 is considered a small effect, 0.5 a medium effect and 0.8 a large effect [30].

Differences in PA and SB by: sex, BMI, weight status, NOS, NOS over and under 53, family group, first-born status, full time or part-time preschool attendance and parental education were calculated using univariate ANOVA. Eta squared was calculated to assess effect size. An effect size of 0.1 is considered small, 0.25 medium and 0.4 large [30].

To further explore the data the lowest and highest quartiles of NOS and BMI were examined in relation to the lowest and highest quartiles of PA and SB. Additionally, NOS was examined with respect to parental weight status. As these analyses did not result in any additional findings compared to those using total score and dichotomy variables the results are not reported.

P-values <0.05 were considered statistically significant. Version 22 of SPSS for Windows (SPSS Inc., Chicago, IL) was used for the analyses.

## Results

In total, 178 children attended the two-year visit and 138 of them wore the accelerometer. Of these children, 15 were excluded due to having less than four days of valid data (n = 13) and missing weekend data (n = 2). Thus 123 children remained for analysis. No differences in background characteristics between children who wore and children who did not wear the accelerometer were found, except for BMI, which was 16.6 (SD 2.6) and 17.5 (SD 1.7), respectively (p = 0.03). Data were missing for BMI (n = 3, due to child refusing to have weight or height measured), motor skills (n = 5), child-care situation (n = 14) and parental education (n = 1). However, these children were included in all analysis, excluding only the missing variable. Characteristics of the included children are shown in Table 1.

**Table 1:** **Descriptive characteristics of participants**

	**Mean (SD)**	**n (%)**	**Missing n (%)**
*Age (years)*	2.03 (0.1)		
*Sex*			
Boys		61 (50)	
Girls		62 (50)	
*BMI (kg/m²)*¹	16.9 (1.4)		3 (2)
*BMI SDS*²	-0.2 (1.1)		3 (2)
*Weight status*³			3 (2)
Normal weight		104 (87)	
Overweight		16 (13)	
*Family group* ^*4*^			
High risk		86 (70)	
Low risk		37 (30)	
*NOS* ^*5*^	54.1 (1.9)		5 (4)
*NOS <53*		23 (20)	5 (4)
*First born*		62 (50)	
*Child care*			14 (11)
Preschool full-time		83 (76)	
Preschool part-time		18 (17)	
Other		8 (7)	
*High parental education*		90 (74)	1 (1)

¹BMI = Body Mass Index.

²BMI SDS = Body Mass Index Standard Deviation Score.

³BMI categories according to Cole et al.

^4^Family group based on parental BMI. High risk: both parents BMI ≥25 or at least one parent ≥30.

^5^NOS = Neurological Optimality Score.

Total sample N=123.

Descriptive data on PA and SB are presented in Table 2. Children performed low-intensity PA for 34% and were active at a high-intensity 11% of the day. On average 55% of the day was spent being sedentary. Of the 123 children, 97 (79%) had any bout of 5 minutes in low-intensity PA. Four children (3%) had any bout of 5 minutes in high-intensity PA. All children had one or more bouts in sustained SB (mean 2.5, SD 1.2) lasting for 30 minutes or more. Weekdays and weekend days were compared and found to be almost similar. Number of steps per day and time spent in low-intensity PA differed: on weekdays, 363 more steps (p = 0.01, Cohen’s d = 0,19) and six more minutes in low-intensity PA (p = 0.04, Cohen’s d = 0,16). The increase in low PA seems to be taken from a decrease in both high-intensity PA and in SB. No other significant differences between weekdays and weekend days were found for any PA or SB variable (p = 0.2–0.86, Cohen’s d < 0.07).

**Table 2:** **Physical activity and sedentary behavior variables (mean (SD) per day)**

		**Sex**			**Weight status**			**NOS** ^**4**^		
	**Total sample (n = 123)**	**Boys (n = 61)**	**Girls (n = 62)**	**P**	**Normal weight (n = 104)**	**Overweight (n = 16)**	**P**	**<53 (n = 23)**	**≥53 (n=95)**	**p**
*Average PA¹ (CPM² vertical)*	1814 (391)	1812 (368)	1816 (416)	0.95	1817 (374)	1841 (506)	0.82	1922 (443)	1797 (377)	0.17
*Average PA¹ (CPM² VM³)*	3046 (524)	3003 (547)	3087 (501)	0.38	3041 (526)	3163 (488)	0.38	3236 (541)	3017 (506)	0.07
*Steps*	11152 (1628)	11181 (1733)	11124 (1531)	0.85	11175 (1649)	11251 (1422)	0.86	11527 (1698)	11114 (1581)	0.27
*Sedentary*										
Minutes	432 (47)	436 (45)	428 (49)	0.38	432 (48)	422 (40)	0.42	416 (43)	434 (47)	0.09
No of 30 min bouts	2.5 (1.2)	2.5 (1.3)	2.4 (1.0)	0.66	2.2 (1.3)	2.4 (0.8)	0.73	2.4 (1.3)	2.5 (1.2)	0.67
Total time in 30 min bouts	153 (78)	151 (83)	155 (75)	0.78	155 (83)	146 (51)	0.63	143 (67)	157 (83)	0.44
*Low PA*										
Minutes	265 (33)	263 (30)	267 (36)	0.56	265 (34)	270 (26)	0.59	275 (29)	264 (34)	0.15
No of 5 min bouts	2.1 (1.5)	2.3 (1.6)	1.9 (1.4)	0.21	2.2 (1.5)	1.9 (1.4)	0.44	2.1 (1.4)	2.1 (1.5)	0.84
Total time in 5 min bouts	12 (8)	13 (9)	11 (8)	0.14	12 (9)	10 (8)	0.40	11 (8)	12 (9)	0.72
*High PA*										
Minutes	84 (23)	82 (25)	86 (21)	0.36	84 (23)	89 (23)	0.83	90 (26)	83 (22)	0.18
No of 5 min bouts	0.2 (0.3)	0.2 (0.3)	0.2 (0.3)	0.89	0.2 (0.3)	0.2 (0.3)	0.93	0.2 (0.3)	0.2 (0.3)	0.66
Total time in 5 min bouts	2 (13)	1 (2)	3 (18)	0.30	2.5 (14.0)	1.0 (1.6)	0.68	1.3 (2.0)	1.1 (1.7)	0.63

¹PA=Physical activity.

²CPM=Counts per minute.

³VM=Vector Magnitude.

^4^NOS=Neurological Optimality Score.

The level and pattern of PA and SB remained similar across sex, BMI and NOS (Table 2). No significant differences were identified for any PA or SB variable by sex, BMI, NOS (as categorical and as continuous variable) or any family-related variable (p = 0.07–0.95, eta square <0.03).

## Discussion

To the best of our knowledge this is the first study on two-year-old children to objectively examine PA and SB over several days. The most important findings were that the children spent about half of their daytime being active, at either a high or low intensity. Further, neither sex, BMI, motor skills, nor family-related factors were correlated with two-year-old children’s PA or SB.

The finding that 35% of sedentary time consisted of sustained sitting for at least 30 minutes together with the finding that bouts lasting longer than five minutes in high intensity PA were very rare confirms the assumption that young children have an intermittent activity pattern [14]. Thus, PA data based on proxy reports could be inaccurate. Objective measures, such as accelerometry used in this study, can capture short bursts of PA and SB and should be the method of choice for this population [14].

In studies on school-aged children results are often compared with WHO guidelines recommending children to be physically active at least 60 minutes per day [31]. The evidence to support the guidelines for very young children is insufficient; however some countries have developed guidelines for children under the age of five [32,33], recommending that very young children engage in PA for at least 180 minutes per day. However, no recommendations regarding intensity level are provided. The children in the present study were physically active for about 350 minutes per day, when both low and high intensity were considered together.

Swedish school-aged children, six years of age and older, appear to be less active on weekends compared to weekdays [6]. In our sample, weekdays and weekend days were almost identical, except that on weekdays the children took significantly more steps and spent more time in low-intensity PA, which may indicate that the activities of daily living are already slightly different on weekdays and weekends for this age group. Most Swedish two-year-old children, like the children in our sample, attend preschool on weekdays, allowing them many opportunities to be physically active both indoors and outdoors. On weekend days, however, the opportunities to be active may be different. On weekend days child PA may be affected by parental PA. This was not investigated here but should be examined in future studies. However, the difference was small: only about 350 steps and six minutes in low-intensity PA per day and the effect size was low. Since there were no differences in average PA, and both time in high-intensity PA and sedentary time were lowered while low-intensity PA was increased on weekdays, this difference is likely not relevant from a clinical perspective. Since almost all children (76%) in our sample attended preschool full-time, it was not possible to draw any further conclusions from analyses related to child-care.

The Early STOPP study involves two groups with regard to parental weight status and is therefore optimized for the detection of differences associated with parental weight. Despite that, no differences in any PA or SB variable or in motor skills were found with regard to weight status or BMI of children or parents. This result are in line with previous studies showing that parental obesity does not seem to affect early weight gain or eating patterns [19,34]. However, within the Early STOPP cohort we have shown that weight at one year of age is inversely associated with parental education [19]. Other groups have also reported that other markers of socioeconomic status are associated with early weight gain [35,36]. Since in the present study PA is not associated with parental education it is likely that food intake patterns are more important than PA or socioeconomic status for early obesity development.

No correlates to any PA or SB variable were found despite the fact that the individual variation in PA was pronounced and similar to what we have found in older children [6]. The PA for children two years of age seems to be primarily regulated by endogenous factors such as genetic and epigenetic variations, although exogenous factors that remain to be identified probably also contribute. Only one previous study on correlates of objectively measured PA and SB among two-year-old children has been found in the literature [37]. In that study PA was measured by a hip-worn accelerometer for two days. In contrast to our findings, in this age group, being male was already positively associated with time spent in moderate-to-vigorous PA and negatively associated with time spent sedentary. As in the present study the child’s BMI and parental educational level was not correlated to PA. Due to lack of studies on children under the age of three further comparisons are difficult to make. It is yet unclear if PA is associated to sex [12,38,39], child’s BMI [38,39], child adiposity [40], parental weight [12] or parental education [5,41] in preschool children, that is, three-five-year-olds. Motor skills have been examined in relation to objectively measured PA and SB in some studies on four-five year olds [8,12,42-44]. Overall, the results are unclear but some studies support a positive association between PA and gross motor skills [8,42].

The accelerometers were not removed at night and to exclude sleep time the hours between 8 p.m. and 7 a.m. were deleted. We did not consider day time naps. Thus, time when the child was sleeping outside the removed hours was considered sedentary time and if the child was awake and active at night, that activity was disregarded. However, it is not likely that a few minutes of missing activity or sedentary time would affect the results.

Strengths of the present study are its use of an objective method to measure PA and SB over several days. We used age and site specific intensity thresholds to categorize accelerometer data as time spent on different intensity levels. Further, motor skills, height and weight were also measured objectively, thus eliminating bias associated with proxy reports.

One limitation is that the included children were homogenous with regard to the included correlates, such as motor skill and BMI. Most children had high scores on the motor-skill test. The motor-skill test used is developed to capture minor differences in motor skill development and was considered the best method of choice. However, it has mainly been used on in vitro fertilized children and on children prenatally exposed to dioxins, and may thus not have been sufficiently sensitive. The accelerometer was worn on the wrist, which might be a limitation. Placement site of the monitor will affect the output, making comparison of average PA (counts/minute) across studies with different placement sites impossible. However, we used age and site-specific intensity thresholds to calculate time in different intensities, enabling comparison of time in sedentary, low and high intensity PA. Step counts from a wrist worn Actigraph have not been validated for children under the age of 3. Thus, we are not sure the number of steps measured is actually true steps. We chose to include information about steps, in addition to average activity and time in intensities, as it might be a proxy for overall activity. Despite the high number of overweight and obese parents as a consequence of the study design, only a few of the children were considered overweight. Further, children who did not wear the accelerometer had a higher BMI than those included in the study.

## Conclusions

In conclusion this study demonstrates that two-year-old children have an intermittent activity pattern, with only slight variation over weekdays, and spend about half of their days being sedentary. In addition no correlates to children’s PA or SB were found. Taken together, the results indicate that PA or SB in two-year old children is not related to being overweight but these results needs to be confirmed by future prospective studies.

## Abbreviations

BMI: Body mass index
BMI: SDS body mass index standard deviation score
CPM: Counts per minute
NOS: Neurological optimality score
PA: Physical activity
SB: Sedentary behavior
SD: Standard deviation
VM: Vector magnitude
